# Causal relationship between non-alcoholic fatty liver disease and sarcopenia: a bidirectional Mendelian randomization study

**DOI:** 10.3389/fmed.2024.1422499

**Published:** 2024-09-18

**Authors:** Meng Chen, Jili Liu, Xin Xia, Yarong Wang, Hongying Zheng

**Affiliations:** ^1^Department of Geriatrics and Special Needs Medicine, General Hospital of Ningxia Medical University, Yinchuan, China; ^2^Department of Geriatrics, The First Hospital, Shanxi Medical University, Taiyuan, China; ^3^The Center of Gerontology and Geriatrics and National Clinical Research Center for Geriatrics, West China Hospital, Sichuan University, Chengdu, China

**Keywords:** non-alcoholic liver disease, sarcopenia, Mendelian randomization analysis, causal relationship, genetics

## Abstract

**Introduction:**

A correlation between non-alcoholic fatty liver disease and sarcopenia is demonstrated, but the causality remains unclear. Our study aims to clarify the point of genetics between non-alcoholic fatty liver disease (NAFLD) and sarcopenia at the level of gene prediction through two-sample Mendelian randomization (MR) analysis.

**Methods:**

The study employed the two-sample MR approach to investigate the bi-directional causality between NAFLD and sarcopenia. Published summary statistics were used to obtain instrumental variables (IVs) at the genome-wide significance level.

**Results:**

IVW analysis showed that the risk of NAFLD was reduced when walking pace was increased (OR = 0.435, 95%CI 0.240–0.789, *p* = 0.006); Increasing appendicular lean mass (ALM) decreased the risk of NAFLD (OR = 0.906, 95%CI 0.838–0.980, *p* = 0.014); Those older than 60 were more likely to suffer from NAFLD if they had low grip strength (OR = 1.411, 95%CI 1.087–1.830, *p* = 0.0012). In the reverse MR study, weight median analysis showed that NAFLD caused a decrease in ALM (OR = 0.953, 95%CI 0.957–0.994, *p* = 0.001); whereas NAFLD showed no correlation with usual walking pace or grip strength (all with *p* > 0.05). MR-Egger regression analysis showed that there was no horizontal pleiotropy in the SNPs (all with *p* > 0.05).

**Conclusion:**

The characteristics related to sarcopenia (usual walking pace, appendicular lean mass and low hand grip strength) may play a causal role in the development of nonalcoholic fatty liver disease, although the underlying mechanisms need to be further investigated. The presence of specific single nucleotide polymorphisms (SNPs) such as rs3747207, rs429358, and rs73001065 has been identified in the PNPLA3, APOE, and MAU2 proteins. These genetic markers represent potential targets for future interventions aimed at addressing, managing, or mitigating the risk of NAFLD.

## Introduction

Non-alcoholic fatty liver disease (NAFLD) is a liver condition characterized by excessive fat accumulation in liver cells, unrelated to excessive alcohol consumption or other identifiable liver damage factors. A systematic review and meta-analysis by Younossi et al. (1) on the epidemiology and natural history of NAFLD showed a global prevalence of 25.2% from 1989 to 2015. Another systematic review and meta-analysis by Riazi et al. (2) indicated that the global prevalence of NAFLD increased from 25.5% before 2016 to 37.8% after 2016, with an estimated overall global NAFLD prevalence of 32.4% and an estimated incidence rate of 46.9 per 1,000 person-years. These findings suggest that the current global prevalence and incidence rates of NAFLD are significantly higher than expected, making NAFLD the most common chronic liver disease globally. With the aging population and the increasing prevalence of metabolic syndrome, it is expected to continue rising at a remarkable pace over the future (3). Sarcopenia is an age-related skeletal muscle disorder characterized not only by decreased muscle mass but also by a decline in muscle strength and physical function (4). In 2010, IWGSOP2 defined sarcopenia as a progressive and systemic skeletal muscle disorder associated with aging. Both the EWGSOP2 and AWGSOP2 have indicated its adverse outcomes are associated with increased risks of falls, fractures, physical disability, and mortality (5, 6). Multiple studies (7–12) have indicated that NAFLD and sarcopenia share some common pathophysiological mechanisms, including obesity, insulin resistance (IR), vitamin D deficiency, aging, lack of physical activity, chronic inflammatory response, and specific cytokines (hepatic and myogenic factors), among others. There is evidence of correlation between NAFLD and sarcopenia, and they May mutually promote each other, acting as risk factors for one another. Although increasing attention has been paid to the relationship between NAFLD and sarcopenia, with substantial support from literature, it remains unclear whether NAFLD is the cause or consequence of sarcopenia, requiring further research to elucidate.

Mendelian randomization (MR) is an effective method for inferring potential causal relationships between exposure and outcome, which can reduce biases caused by confounding factors and reverse causation in epidemiological studies (13). In this study, we employed the two-sample MR method using instrumental variables (IVs) from Genome-Wide Association Study (GWAS) datasets to analyze the causal relationship between NAFLD and sarcopenia. This analysis aimed to evaluate the potential causal effects between NAFLD and sarcopenia-related traits, providing new evidence for understanding their relationship.

## Materials and methods

### Study design

**Figure 1:**
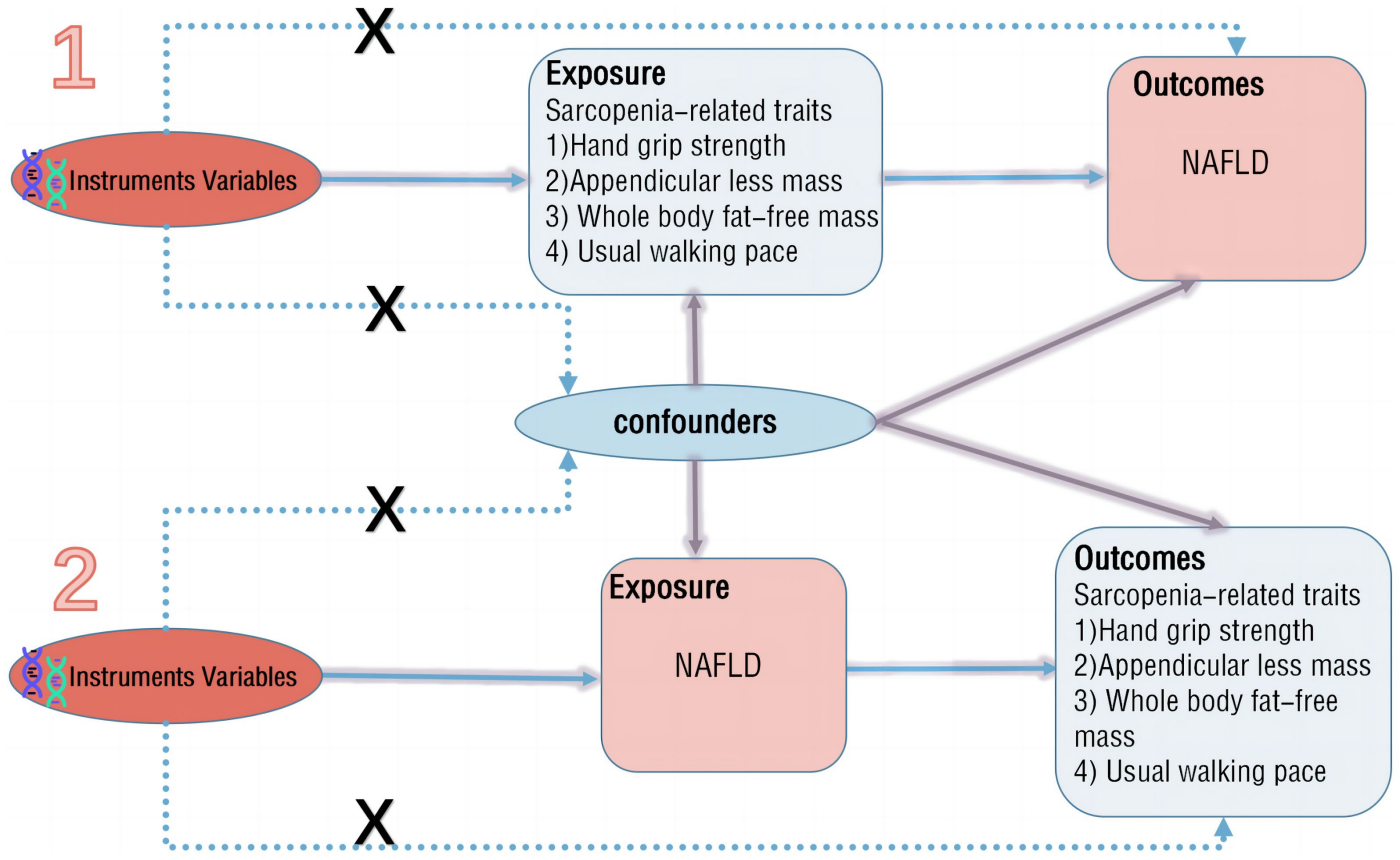
Flowchart of MR analysis.

The aim of this study is to assess the causal relationship between NAFLD and sarcopenia. A two-sample bidirectional MR method is employed, where NAFLD and sarcopenia-related traits serve as both exposure and outcome factors. Initially, in the forward MR analysis, NAFLD is considered as the exposure, with its potential single nucleotide polymorphisms (SNPs) identified for a single-sample MR analysis, while sarcopenia-related traits are treated as outcomes for two-sample MR analysis. Subsequently, in the reverse MR analysis, sarcopenia-related traits are regarded as the exposure, with NAFLD as the outcome for two-sample MR analysis (Figure 1). The study adheres to three main assumptions: (1) IVs are closely associated with the exposure; (2) IVs are independent of any confounding factors influencing the exposure-outcome link; (3) IVs only affect the outcome through the exposure (13).

### Data sources

#### Non-alcoholic fatty liver disease dataset

The dataset utilized for the study of NAFLD is derived from the publicly accessible GWAS summary dataset compiled by Ghodsian et al. (12). This dataset comprises summary statistics from GWAS cohorts sourced from eMERGE and FinnGen, as well as updated NAFLD GWAS data from the UK Biobank (2,558 cases and 395,241 controls) and a newly conducted GWAS in the Estonian Biobank (4,119 cases and 190,120 controls). In total, the dataset includes 8,434 NAFLD cases and 770,180 controls, along with information on 6,784,388 SNPs. Notably, all individuals included in the dataset are of European ancestry, and there is no overlap between the samples.

#### Sarcopenia dataset

The characteristics related to sarcopenia include appendicular lean mass (ALM), grip strength, and usual walking pace, which, respectively, reflect muscle mass, muscle strength, and mobility. ALM has been proposed as an effective and reliable indicator of muscle mass in older adults (14). ALM was quantified by bioelectrical impedance analysis (BIA) of 450,243 individuals from the UK Biobank^1^ comprising 205,513 males and 244,730 females of European ancestry (15). Grip strength is considered an important indicator for diagnosing sarcopenia (5). Grip strength data were sourced from the UK Biobank, including 461,089 right-hand grip strength and 461,026 left-hand grip strength measurements from individuals of European ancestry (16). Additionally, we collected data from the CHARGE consortium, including 256,523 elderly individuals (aged 60 and above) of European ancestry. Jones et al. (17) recorded summary statistics from 22 independent cohorts for maximum grip strength, including the UK Biobank, the US Health and Retirement Study, and the Framingham Heart Study, among others. Summary statistics for usual walking pace were also obtained from the UK Biobank, comprising 459,915 individuals of European ancestry and 9,851,867 SNPs. Here is a summary of the essential information regarding the enrolled traits in Table 1.

**Table 1:** All GWAS datasets selected in this article.

Trait	GWASId	Sample size	Number of SNPs
NaFLD	ebi-a-GCST90091033	778614	6784388
usual walk pace	ukb-b-4711	459915	9851867
Appendicular lean mass	ebi-a-GCST90000025	450243	18071518
Left Hand grip strength	ukb-b-7478	461026	9851867
Right Hand grip strength	ukb-b-10215	461089	9851867
Low hand grip strength (60 years and older)	ebi-a-GCST90007526	256523	9336415

#### Filter instrument variables

According to the research hypothesis, we will search the GWAS database for SNP selection. The selected instrumental variable SNPs should meet the following criteria (18):

1. SNPs that are strongly correlated with the relevant characteristics (with a threshold of *p* < 5 × 10^-8).
2. Clustering based on the linkage disequilibrium (LD) structure of the 1,000 Genomes Project to remove SNPs that are not related to other potential confounding factors, and using SNPs (with an r^2 < 0.001 and a region width of 10,000 kD) to remove LD.
3. Selection of SNPs with an F-statistic >10 to maximize the exclusion of weakly correlated instrumental variables. All the results of F-statistics and *p* values for included SNPs had been listed in Table 1.

Based on these criteria, we will search and select SNPs from the GWAS database that meet the requirements to serve as our instrumental variables.

#### MR statistics

In this study, we utilized the TwoSampleMR package (version 0.5.8) in R (version 4.3.2) to evaluate the mutual influence between NAFLD and sarcopenia-related traits using the inverse variance weighting (IVW) method, weighted median method, and MR-Egger. An odds ratio (OR) and 95% confidence interval (CI) for exposure IVs on outcome IVs were estimated with a *p* < 0.05 considered statistically significant. IVW can provide accurate estimates if all SNPs are valid tool variables (19). When there are no weak IVs, the IVW method is the primary statistical approach, while the other methods are supplements. Additionally, the MR-Egger regression was employed to identify SNP-level pleiotropy and correct for multiple effects. As for directional pleiotropy analyses, we used MR-Egger regression methods to evaluate the possible pleiotropic effect based on the intercept of the model (P for intercept<0.05) (20). Furthermore, leave-one-out analysis was performed using the IVW method to assess whether the overall estimate was influenced by individual SNPs. Cochran Q statistics and MR-Egger regression were used to calculate heterogeneity in the IVW method, and the *p* value was 0.05, indicating that there was a large heterogeneity.

## Results

### Characteristic related to sarcopenia as exposure MR analysis

**Figure 2:**
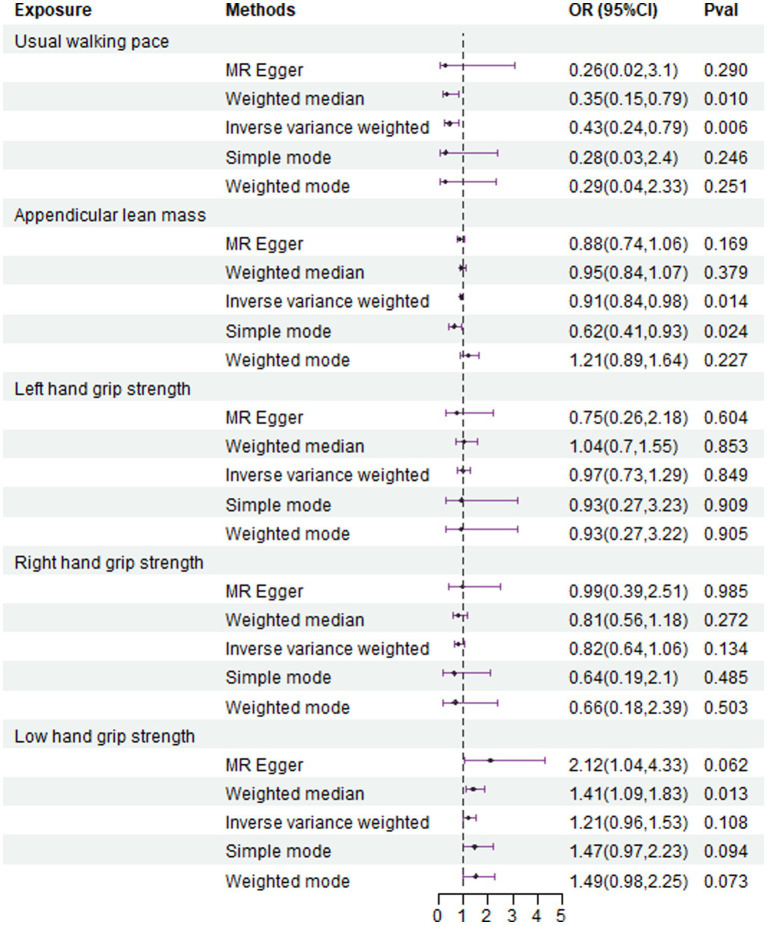
Association between genetical causes of sarcopenia and NAFLD from the UK trait.

**Figure 3:**
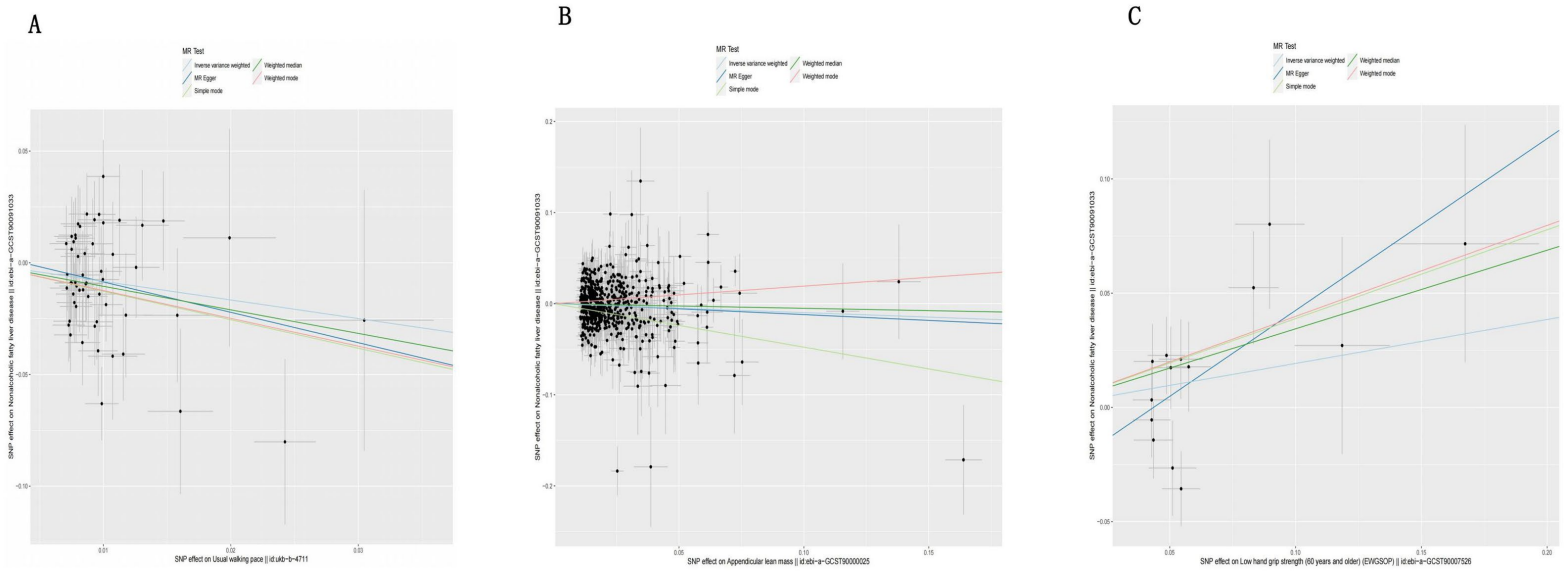
Scatter plots for MR analyses of the causal effect sarcopenia on NAFLD based on UK trait. **(A)**. Usual walking pace **(B)**. ALM **(C)**. Low hand grip (60 years and older). Analyses were conducted using the conventional IVW, WMM and MR-Egger. The slope of each line corresponding to the estimated MR effect per method.

The IVW analysis results indicate that an increase in usual walking pace is associated with a decreased risk of NAFLD (OR = 0.435, 95% CI 0.240–0.789, *p* = 0.006) (Figure 2). An increase in ALM suggests a decreased risk of NAFLD (OR = 0.906, 95% CI 0.838–0.980, *p* = 0.014) (Figure 2). Low grip strength in individuals aged 60 and above is associated with an increased risk of NAFLD (OR = 1.411, 95% CI 1.087–1.830, *p* = 0.0012), as shown in Figure 2. However, levels of left and right grip strength are not associated with the risk of NAFLD (left grip strength OR = 0.973, 95% CI 0.732–1.293, *p* = 0.849; right grip strength OR = 0.823, 95% CI 0.637–1.062, *p* = 0.849) (Figure 2). IVW, weighted median method, and MR-Egger regression analysis results all indicate that left and right grip strength are not associated with the risk of NAFLD (*p* > 0.05); The analysis using the MR-Egger regression method shows that there is no evidence of horizontal pleiotropy for SNPs strongly associated with usual walking pace, ALM, and low grip strength in individuals aged 60 and above in relation to NAFLD (intercepts are 0.005, 0.001, and-0.023 respectively, with *p*-values of 0.673, 0.755, and 0.288). Leave-one-out analyses for these two traits suggested that the estimated causal effects were not significantly influenced by any single instrumental variable. Scatter plots depicting the MR analyses of the causal effects of sarcopenia on NAFLD with statistical significance are presented in Figure 3 (A for usual walking pace, B for AML, and C for 60 older low grip strength, respectively). All the involved funnel plots, scatter plots and “leave-one out analysis” plots in assessing the association between sarcopenia and NAFLD in UK trait were shown in Supplementary file 1.

### Non-alcoholic fatty liver disease as exposure reverse dual sample MR analysis

**Figure 4:**
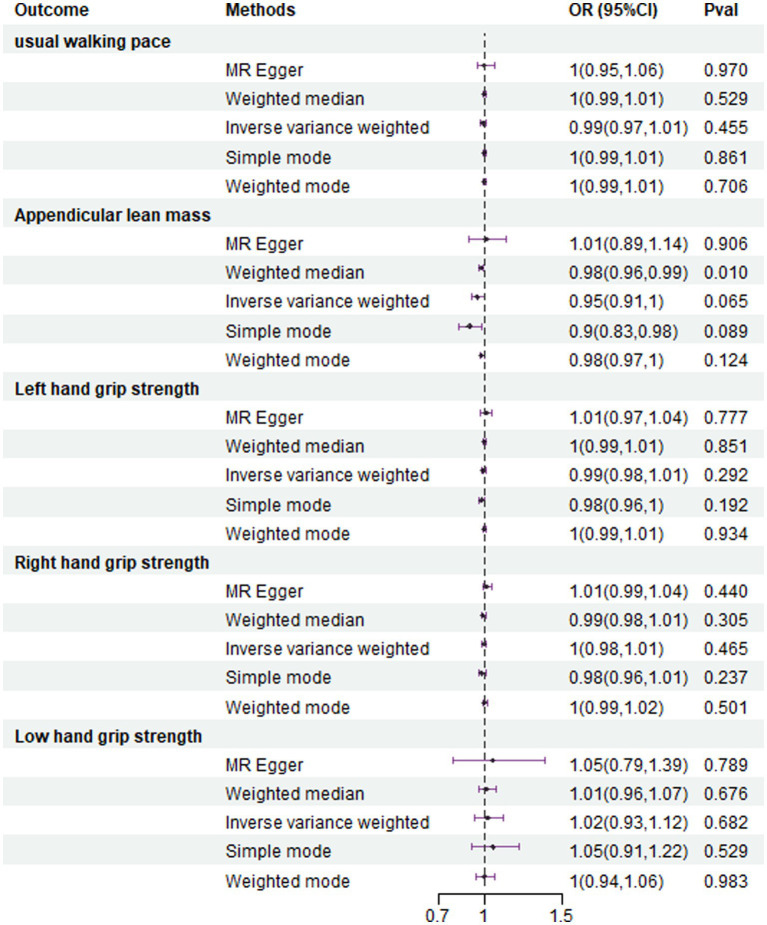
In the reverse MR analysis, association between genetical causes of NAFLD and sarcopenia from the UK trait.

**Figure 5:**
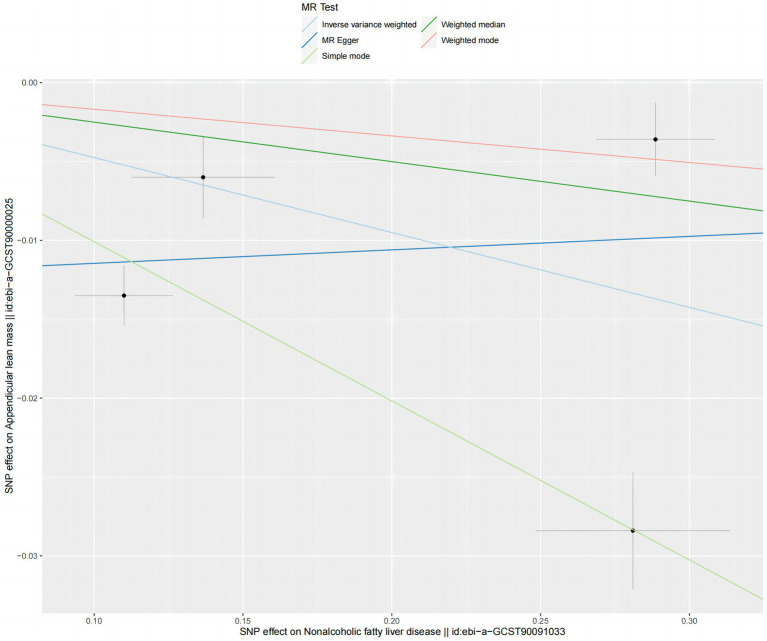
Scatter plots for MR analyses of the causal effect NAFLD on ALM based on UK trait. Analyses were conducted using the conventional IVW, WMM and MR-Egger. The slope of each line corresponding to the estimated MR effect per method.

The analysis using the weighted median method shows a more pronounced association between NAFLD and ALM (OR = 0.953, 95% CI 0.957–0.994, *p* = 0.001) (Figure 4). However, NAFLD is not significantly associated with usual walking pace (OR = 0.993, 95% CI 0.794–1.012, *p* = 0.455) (Figure 4), grip strength (left grip strength OR = 0.993, 95% CI 0.981–1.006, *p* = 0.292; right grip strength OR = 0.994, 95% CI 0.983–1.008, *p* = 0.464) (Figure 4), and low grip strength in individuals aged 60 and above (OR = 1.02, 95% CI 0.927–1.123, *p* = 0.682) (Figure 4). Additionally, no evidence of horizontal pleiotropy is found for the four SNPs strongly associated with NAFLD and ALM (intercept is −0.012, with a *p*-value of 0.438). Scatter plots illustrating the MR analyses of the causal effects of NAFLD on AML with statistical significance are presented in Figure 5.

### Predictive analysis of genetic correlation

**Figure 6:**
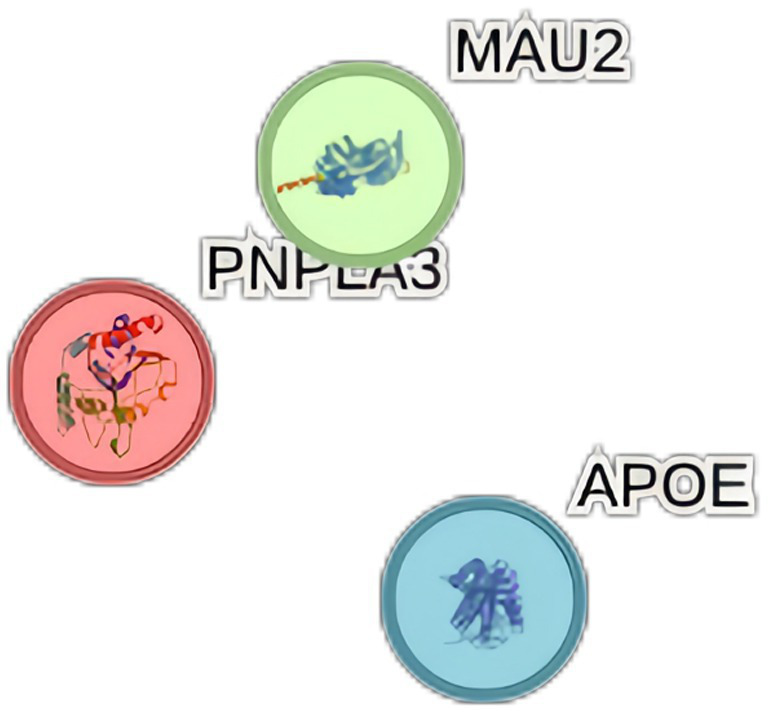
The protein of PNPLA3, APOE, and MAU2.

When NAFLD was considered as the exposure, we conducted gene pathway enrichment analysis on the four SNPs highly associated with NAFLD. Among them, rs3747207, rs429358, and rs73001065 are expressed in PNPLA3, APOE, and MAU2 proteins respectively, it is shown in Figure 6. However, no relevant enrichment pathways were found.

## Discussion

This comprehensive MR study utilized summary statistics from GWAS to conduct a comprehensive evaluation of the causal relationship between genetically predicted NAFLD and sarcopenia. Based on our research findings, we have successfully concluded that sarcopenia and NAFLD may have significant causal effects on each other. Higher gene expression levels of sarcopenia-related traits, including slow usual walking pace, decreased AML, and low grip strength, are correlated with an elevated risk of NAFLD, with a particular emphasis on the significance of reduced grip strength in individuals aged 60 and above. Moreover, genetic expression of NAFLD may substantially heighten the likelihood of developing sarcopenia-related traits, notably decreased appendicular lean mass, although no definitive associations were observed with usual walking pace and grip strength.

Numerous studies have been conducted on the topic, including a bidirectional Mendelian randomization study by Zhao et al. (21) that utilized whole-body lean mass and appendicular lean mass as indicators of sarcopenia to examine their association with NAFLD through GWAS databases. The findings of this study revealed no significant causal relationship between the two variables, aligning with our own results when employing appendicular lean mass as the instrumental variable. In contrast, the weighted median method employed in a separate investigation indicated a potential causal association between NAFLD and ALM. Yuan et al. (22) conducted a Mendelian randomization study utilizing grip strength, appendicular lean mass, and lower limb bioelectrical impedance analysis (BIA) as instrumental variables derived from GWAS data, revealing a negative relationship between NAFLD and appendicular lean mass that aligns with our own research findings. Moreover, numerous cohort studies have also documented comparable results. A cross-sectional study comprising 15,132 individuals with NAFLD found a significant relationship between sarcopenia and NAFLD, independent of obesity or insulin resistance (23), although causality was not definitively established. A separate prospective study involving 309 individuals with biopsy-confirmed NAFLD revealed a connection between decreased muscle mass and the histological severity of NAFLD in this cohort (24).

Although MR studies offer a valuable approach for evaluating causal relationships, they are constrained by limitations pertaining to variable selection, data source quality, and the MR methodology. Consequently, this study is not without its drawbacks. Primarily, MR studies necessitate large sample sizes to guarantee sufficient statistical power. With restricted sample sizes, the statistical power of MR analyses may be diminished, leading to a lack of robustness in the findings. Additionally, it is important to note that SNPs can exhibit varying expressions and impacts on phenotypic traits, including potentially contradictory effects. As such, careful deliberation is essential when choosing suitable instrumental variables. Consequently, further investigation into the functionality of SNPs May be warranted to mitigate these constraints. In the course of conducting gene pathway enrichment analysis, numerous SNPs strongly associated with NAFLD were identified. It may be possible to use these targets as key targets for future efforts to reverse, treat, or even reduce the risk of sarcopenia in the future.

The polymorphic site rs3747207, located on the PNPLA3 gene encoding the Patatin-like phospholipase domain-containing protein 3, is a member of the patatin-like phospholipase family (25). PNPLA3 plays a crucial role in lipid metabolism and is predominantly expressed in hepatocytes and hepatic stellate cells, where it functions as a regulator of lipid droplets (26). Lipid droplets are intracellular vesicular structures responsible for the storage of fatty acids and triglycerides, with the PNPLA3 protein closely involved in lipid droplet metabolism (27). PNPLA3 exerts regulatory control over lipid droplet breakdown and fatty acid release through its phospholipase activity, thereby playing a crucial role in the maintenance of energy balance and lipid metabolism homeostasis. Various studies (26, 28, 29) have identified specific SNPs within the PNPLA3 gene that are linked to the onset and advancement of NAFLD, suggesting PNPLA3 as a promising therapeutic target for further investigation into these conditions.

Moreover, our study has identified a correlation between the SNP rs429358 and NAFLD. Previous research (30) suggests that rs429358 is situated within the APOE gene, which codes for apolipoprotein E (ApoE), a protein closely linked to lipid metabolism and cholesterol transport. rs429358, in conjunction with another SNP rs7412, determines the three primary alleles of the APOE gene: ε2, ε3, and ε4 (31). Polymorphisms in the APOE gene have been linked to an elevated risk of various diseases, such as Alzheimer’s disease and cardiovascular diseases, and even skeletal muscle phenotypes (32, 33). Research indicates that APOE May play a significant role in the development and progression of NAFLD by modulating lipid metabolism, inflammation, oxidative stress, autophagy, and mitochondrial dysfunction (34, 35). Further investigation into the precise mechanisms by which APOE influences NAFLD could offer valuable information for the design of novel therapeutic interventions.

Additionally, our analysis revealed that the SNP rs73001065 is situated in the coding or regulatory region of the MAU2 gene, potentially impacting its activity or expression levels. MAU2, a mitochondrial enzyme known as Mitochondrial Amidoxime, Ureidase 2, participates in multiple metabolic pathways such as the urea cycle and amino acid metabolism. Research (36, 37) findings indicate that MAU2 is predominantly expressed in the liver and May influence serum lipid levels. Its essential function in preserving liver function and metabolic equilibrium underscores its significance. Further investigation is warranted to elucidate the precise mechanisms by which MAU2 contributes to the pathogenesis and progression of NAFLD, as well as the therapeutic advantages of targeting this pathway for enhanced treatment modalities.

## Conclusion

The characteristics related to sarcopenia (usual walking pace, appendicular lean mass and low hand grip strength) may play a causal role in the development of nonalcoholic fatty liver disease, although the underlying mechanisms need to be further investigated. The presence of specific single nucleotide polymorphisms (SNPs) such as rs3747207, rs429358, and rs73001065 has been identified in the PNPLA3, APOE, and MAU2 proteins. These genetic markers represent potential targets for future interventions aimed at addressing, managing, or mitigating the risk of NAFLD.

## Data Availability

The original contributions presented in the study are included in the article/Supplementary material, further inquiries can be directed to the corresponding author.

## Footnotes

^1^https://www.ebi.ac.uk/gwas/publications/33097823
