# Supplementary figures and images for: Causal relationship between non-alcoholic fatty liver disease and sarcopenia: a bidirectional Mendelian randomization study
Source: Front Med (Lausanne). 2024 Sep 18;11:1422499. doi: 10.3389/fmed.2024.1422499 (PMC11445014; doi:10.3389/fmed.2024.1422499)

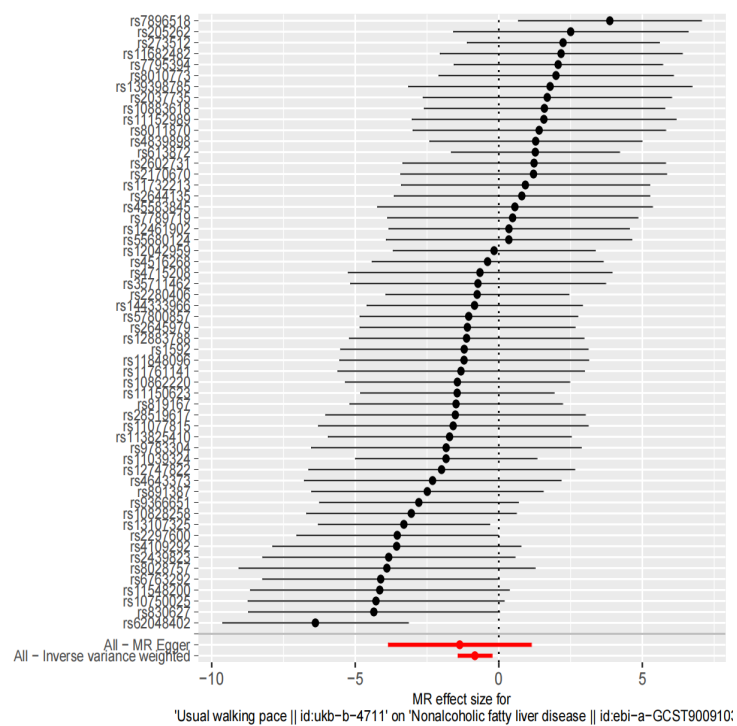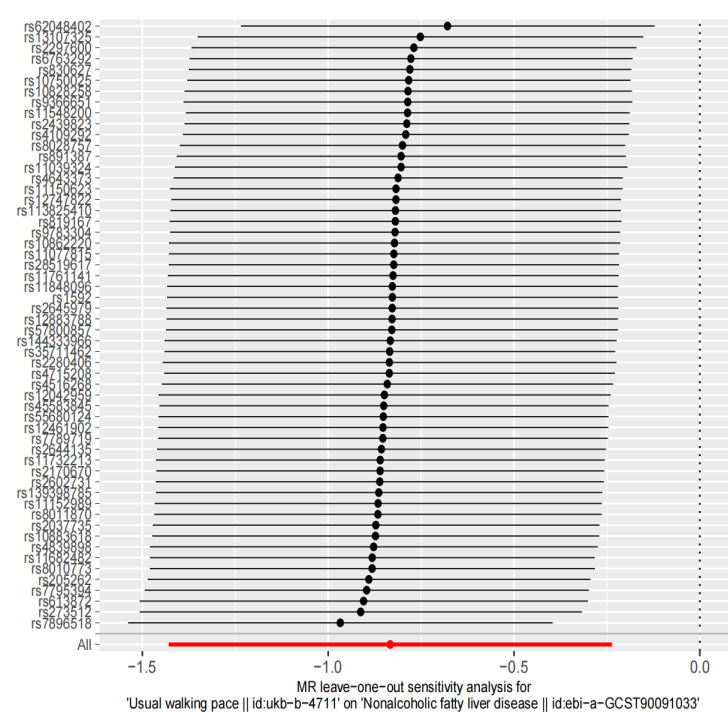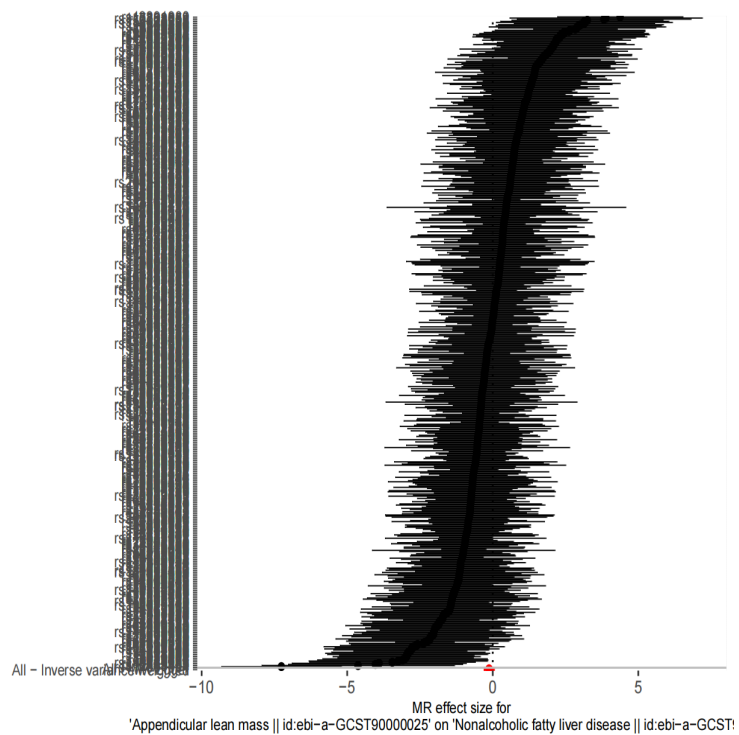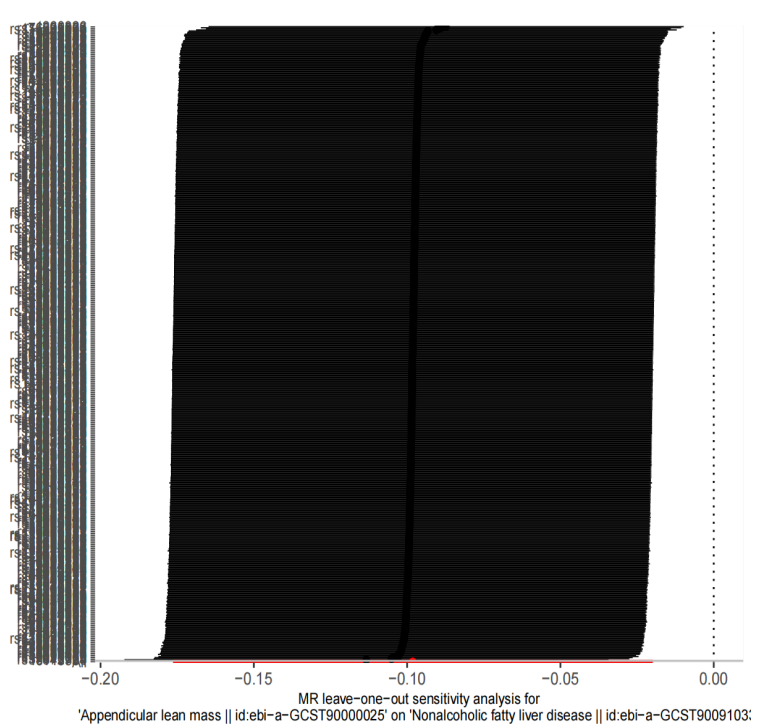

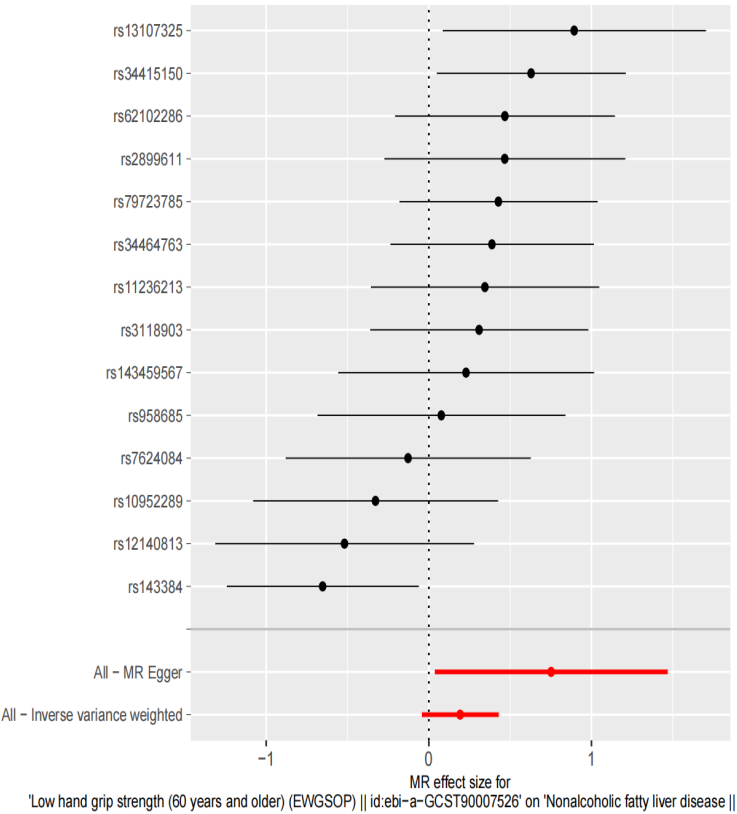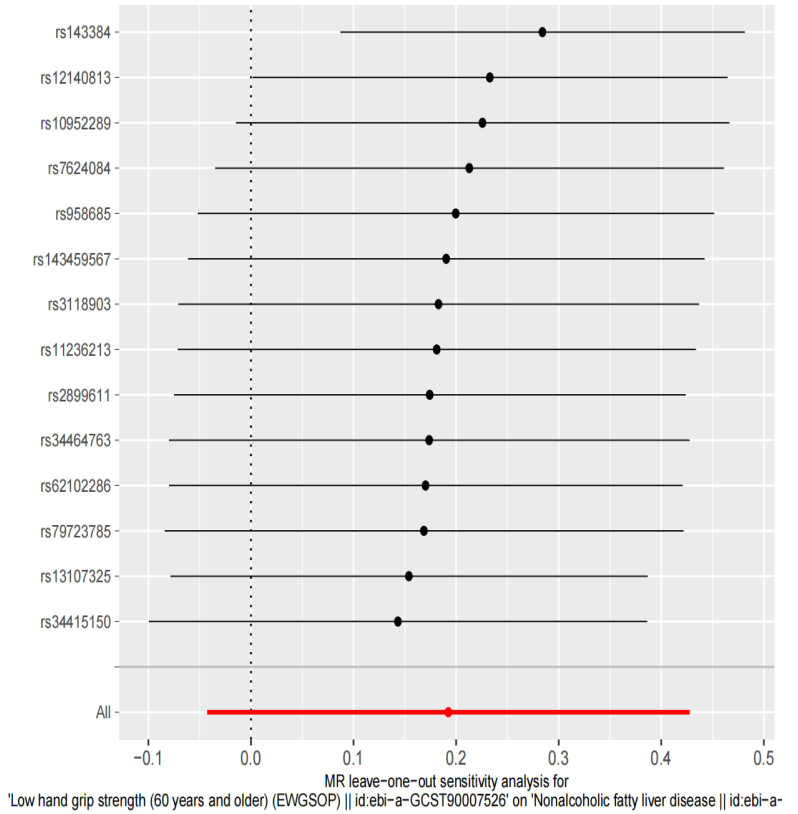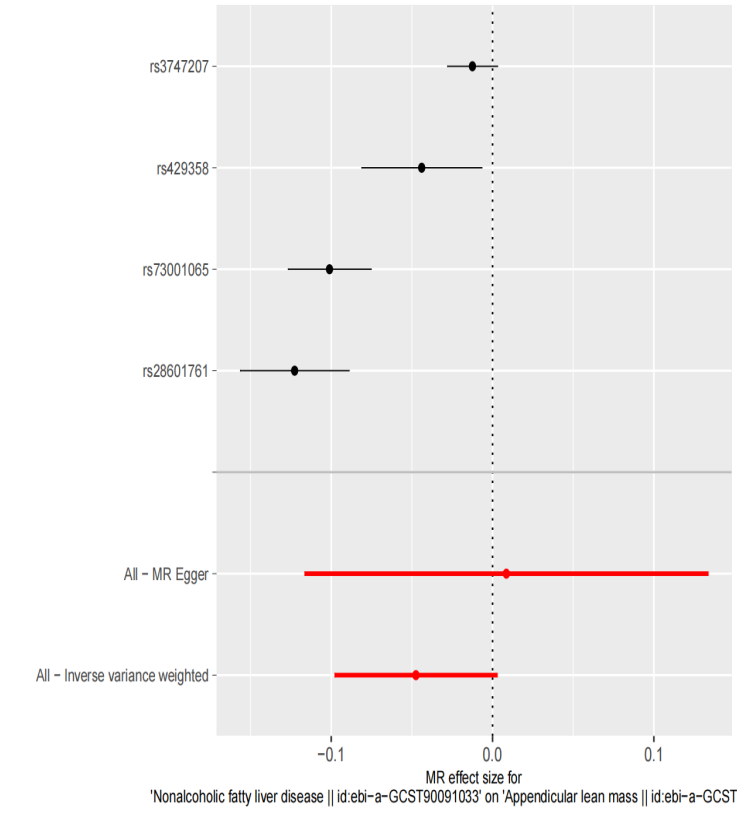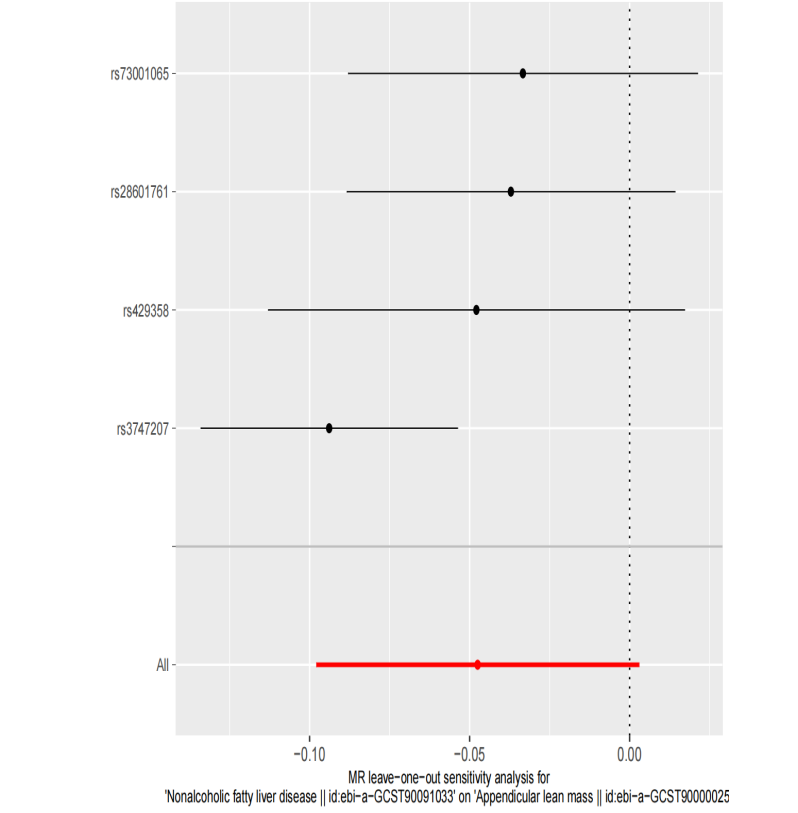

Supplement: Supplementary file 1 [file Data_Sheet_1.PDF]
