# Supplementary material for: Causal relationship between non-alcoholic fatty liver disease and sarcopenia: a bidirectional Mendelian randomization study
Source: Front Med (Lausanne). 2024 Sep 18;11:1422499. doi: 10.3389/fmed.2024.1422499 (PMC11445014; doi:10.3389/fmed.2024.1422499)
Supplement: Supplementary file 2 [file Data_Sheet_2.PDF]

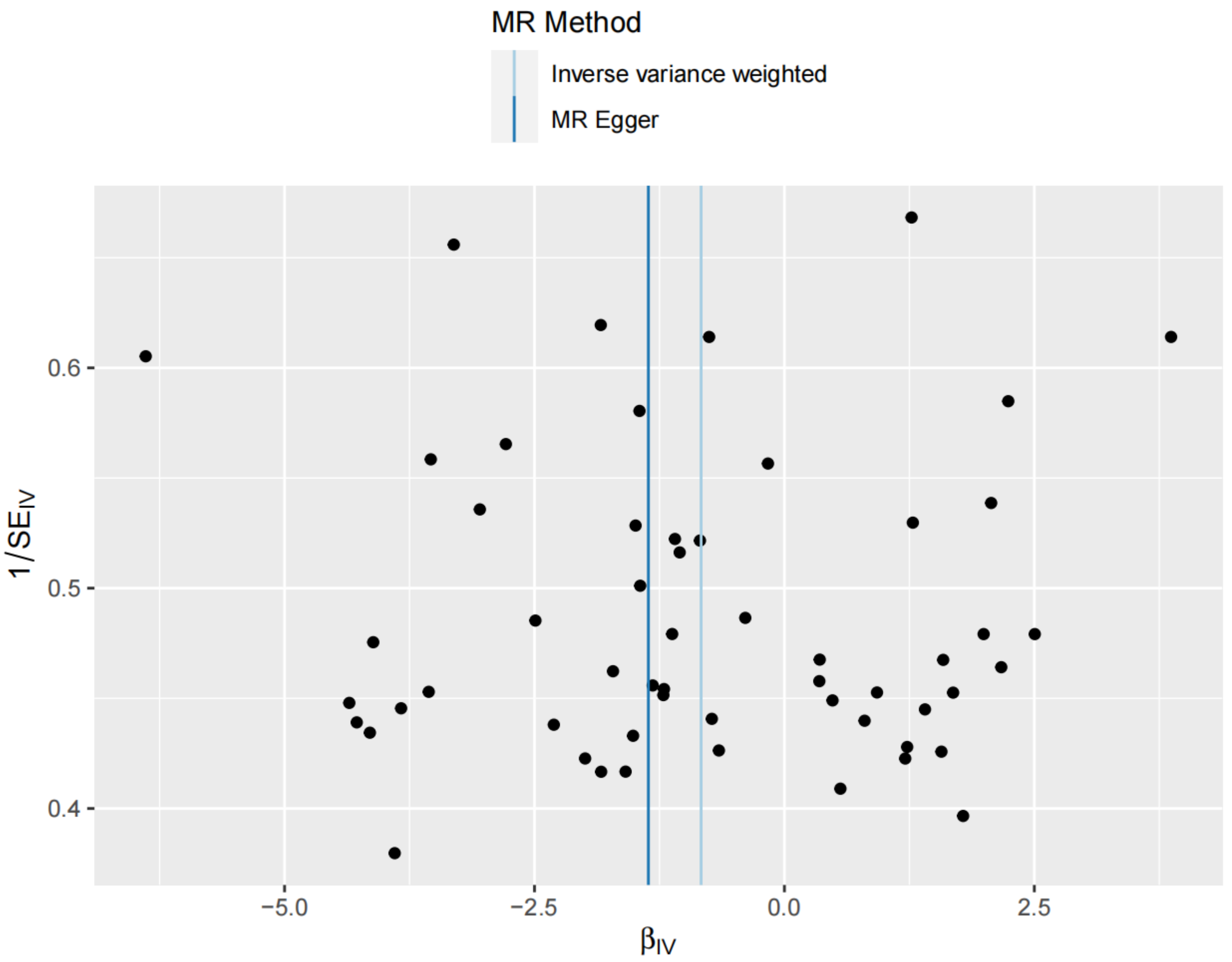

Funnel plots to assess heterogeneity for usual walking pace using all SNPs with the MR Egger and IVW methods

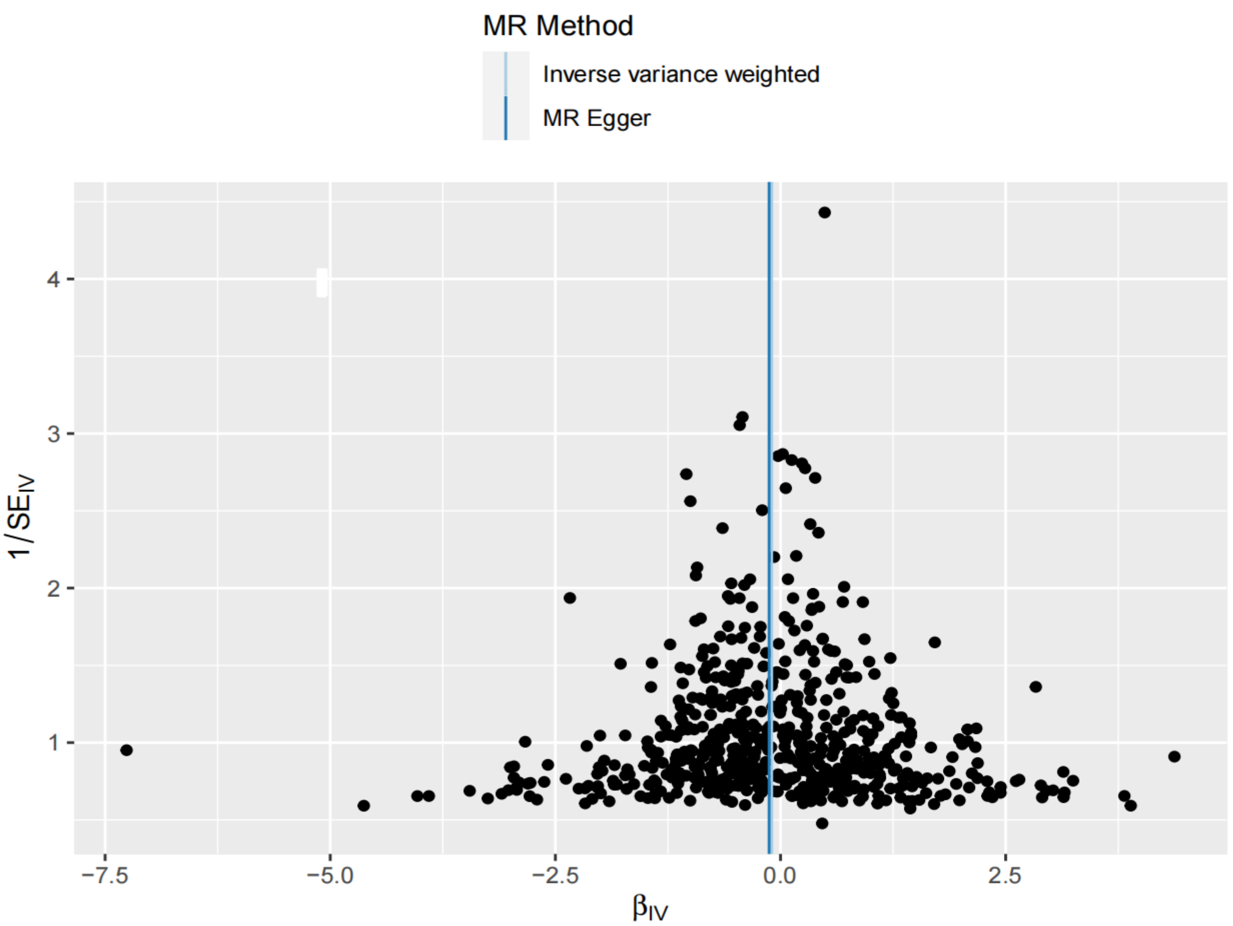

Funnel plots to assess heterogeneity for AML all SNPs with the MR Egger and IVW methods

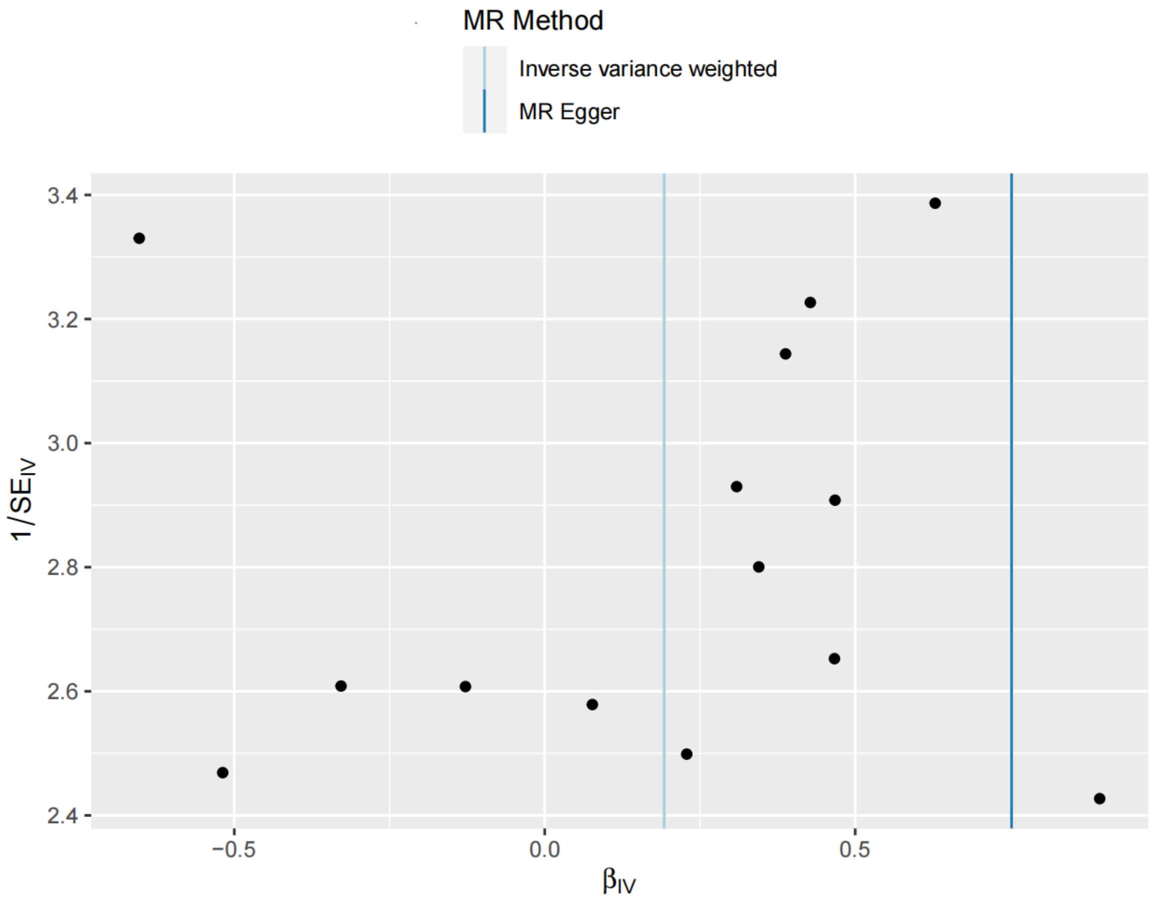

Funnel plots to assess heterogeneity for low hand grip strength using all SNPs with the MR Egger and IVW methods

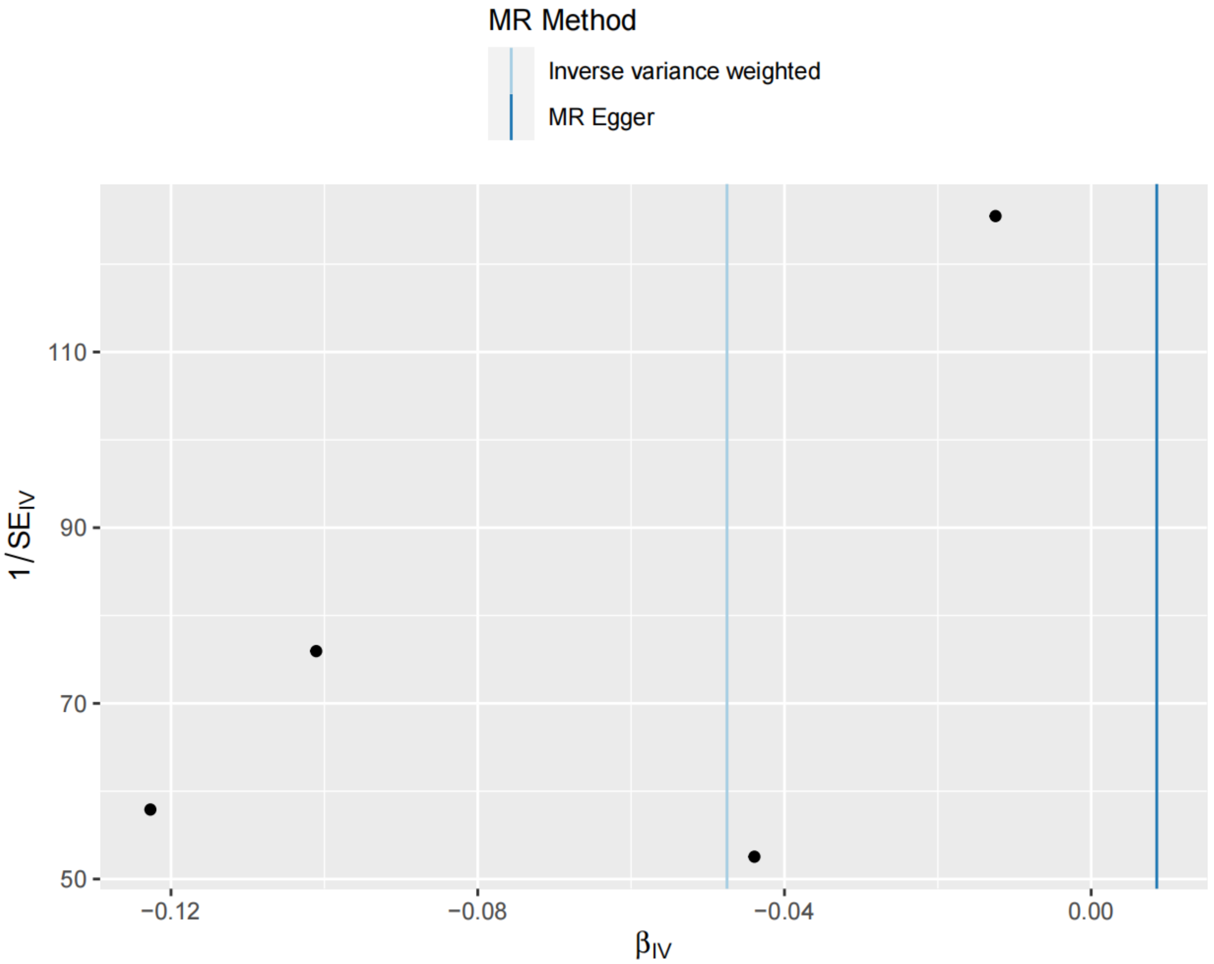

Funnel plots to assess heterogeneity for NAFLD-AML using all SNPs with the MR Egger and IVW methods
