# Supplementary material for: Causal relationship between non-alcoholic fatty liver disease and sarcopenia: a bidirectional Mendelian randomization study
Source: Front Med (Lausanne). 2024 Sep 18;11:1422499. doi: 10.3389/fmed.2024.1422499 (PMC11445014; doi:10.3389/fmed.2024.1422499)
Supplement: Supplementary file 3 [file Table_1.DOCX]

**Table1**

| **Trait** | **GWASId** | **Sample size** | **Number of SNPs** |
| --- | --- | --- | --- |
| **NaFLD** | **ebi-a-GCST90091033** | **778614** | **6784388** |
|  |  |  |  |
| **usual walk pace** | **ukb-b-4711** | **459915** | **9851867** |
|  |  |  |  |
| **Appendicular lean mass** | **ebi-a-GCST90000025** | **450243** | **18071518** |
|  |  |  |  |
| **Left Hand grip strength** | **ukb-b-7478** | **461026** | **9851867** |
|  |  |  |  |
| **Right Hand grip strength** | **ukb-b-10215** | **461089** | **9851867** |
|  |  |  |  |
| **Low hand grip strength**  **(60 years and older)** | **ebi-a-GCST90007526** | **256523** | **9336415** |
|  |  |  |  |
|  |  |  |  |

**All GWAS datasets selected in this article**
